# The Vest Pocket Anatomist

**Published:** 1887-08

**Authors:** 


**The Vest Pocket Anatomist** (*founded upon Gray.*) By C. Henri Leonard, A. M., M. D., Professor of the Medical and Surgical Diseases of Women in the Detroit College of Medicine. Thirteenth revised edition, enlarged by Sections on Anatomical Triangles and Spaces, Herniæ, Gynæcological Anatomy and Dissection Hints. Detroit: The Illustrated *Medical Journal Co.*, 1887, cloth, 86 illustrations, 154 pages, post-paid, 75 cents.

This little volume in its former editions is so well-known that it is only necessary to confine our notice to this, the *thirteenth* edition, which contains very clear and accurate topographical plates of the Venous, Arterial and Nervous Systems, photo-engraved from the English cuts in Gray's Anatomy. This makes the work especially of value to accompany the surgical case of any practitioner that is doing much work in this line, who may wish at his hand a "regional reminder" of the placement of arteries and veins that he may wish to avoid in making his incisions. For this special purpose this little book, since it has the addition of these eighty-six engravings, is of a good deal of value to the country practitioner who sometimes does not have the time to return to his office to consult his more pretentious volumes. The "Dissection Hints" show the incisions to be made in post-mortems to advantage.

